# Proteome Regulation during *Olea europaea* Fruit Development

**DOI:** 10.1371/journal.pone.0053563

**Published:** 2013-01-17

**Authors:** Linda Bianco, Fiammetta Alagna, Luciana Baldoni, Christine Finnie, Birte Svensson, Gaetano Perrotta

**Affiliations:** 1 Italian National Agency for New Technologies, Energy and Sustainable Economic Development (ENEA), TRISAIA Research Center, Rotondella (Matera), Italy; 2 CNR-Institute of Plant Genetics, Perugia, Italy; 3 Enzyme and Protein Chemistry, Department of Systems Biology, Technical University of Denmark, Lyngby, Denmark; Lawrence Berkeley National Laboratory, United States of America

## Abstract

**Background:**

Widespread in the Mediterranean basin, *Olea europaea* trees are gaining worldwide popularity for the nutritional and cancer-protective properties of the oil, mechanically extracted from ripe fruits. Fruit development is a physiological process with remarkable impact on the modulation of the biosynthesis of compounds affecting the quality of the drupes as well as the final composition of the olive oil. Proteomics offers the possibility to dig deeper into the major changes during fruit development, including the important phase of ripening, and to classify temporal patterns of protein accumulation occurring during these complex physiological processes.

**Methodology/Principal Findings:**

In this work, we started monitoring the proteome variations associated with olive fruit development by using comparative proteomics coupled to mass spectrometry. Proteins extracted from drupes at three different developmental stages were separated on 2-DE and subjected to image analysis. 247 protein spots were revealed as differentially accumulated. Proteins were identified from a total of 121 spots and discussed in relation to olive drupe metabolic changes occurring during fruit development. In order to evaluate if changes observed at the protein level were consistent with changes of mRNAs, proteomic data produced in the present work were compared with transcriptomic data elaborated during previous studies.

**Conclusions/Significance:**

This study identifies a number of proteins responsible for quality traits of *cv.* Coratina, with particular regard to proteins associated to the metabolism of fatty acids, phenolic and aroma compounds. Proteins involved in fruit photosynthesis have been also identified and their pivotal contribution in oleogenesis has been discussed. To date, this study represents the first characterization of the olive fruit proteome during development, providing new insights into fruit metabolism and oil accumulation process.

## Introduction

*Olea europaea* is one of the most economically relevant tree crops in the Mediterranean basin. The oil derived from mechanical extraction from the olive drupes is worldwide appreciated for its properties. The peculiar fatty acids composition of olive oil is gaining increasing attention paid to the nutritional and cancer-protective properties [1]. The quality of olive oil is largely determined by the catabolic and anabolic processes taking place during drupe development and ripening. Developing olives undergo dramatic changes in size, composition, color, texture and flavor, accumulating oil in the mesocarp and, at a lower extent, in the seed [2]. The oil content can reach up to 28–30% of the total pulp fresh weight, with the accumulation peak after the onset of ripening. Olive oil is particularly enriched in the monounsaturated fatty acid oleate (18∶1), reaching percentages up to 75–80% of total fatty acids, followed by linoleate (C18∶2), palmitate (C16∶0), stearate (C18∶0) and linolenate (C18∶3). The final acyl composition enormously varies throughout olive fruit development, according to genotype and environmental conditions. Olive drupe mesocarp can accumulate other important metabolites, including polyphenols, carotenoids, chlorophylls, sterols, terpenoids and a wide range of volatile compounds, all directly or indirectly affecting the olive oil quality and aroma [2]. Given the importance of the olive fruit and the nutritional value of its oil, it would be of great interest the comprehension of metabolic changes leading to the biosynthesis of compounds relevant for the quality of both, fruit and oil.

Olive fruit development is a combination of biochemical and physiological events that occur under strict genetic control and influenced by several environmental conditions [3]. It lasts for 4–5 months and includes 5 main phases: I) fertilization and fruit set, characterized by rapid early cell division promoting embryo’s growth (0–30 DAF -days after flowering), II) seed development, a period of rapid fruit growth due to both intense cell division and enlargement involving mainly growth and development of the endocarp (seed/pit), with little mesocarp development (30–60 DAF), III) pit hardening, during which fruit growth slows down as the endocarp cells stop dividing and become sclerified (60–90 DAF), IV) mesocarp development, representing the second major period of fruit growth, due to the mesocarp development mainly by the expansion of preexisting flesh cells, and intense oil accumulation (90–150 DAF), and V) ripening, when the fruit changes from darklime green to lighter green/purple (since 150 DAF) [2].The ripening in fleshy fruits represents the terminal stage of development in which the biochemistry, physiology and structure are developmentally altered to influence appearance, texture, flavor and aroma. Changes typically include: (1) modification of color through the alteration of content and composition of chlorophylls, carotenoids and/or flavonoids; (2) textural modification via alteration of cell turgor and cell wall structure and/or metabolism; (3) modification of sugars, acids and volatiles that affect nutritional quality, flavor and aroma [4].

Comparative proteomics, based on two-dimensional electrophoresis (2-DE) coupled to tandem mass spectrometry, has the potential to screen many metabolic pathways simultaneously for alterations at the protein level. Nowadays, comparative proteomics is becoming attractive to plant biologists as the availability of nucleotide sequences increases, providing new opportunities for protein identification. Actually, the accumulation of nucleic acid data, in parallel to the advancements in sequencing technologies, has permitted the development of more performing methods for the analysis of protein content also for non-model plants [5].

Despite some EST collections from developing olive fruits have recently been established [6], [7], information concerning the proteomic profile of olive drupes during development is still very limited [8].

In this work, a comparative proteomic approach based on 2-DE and MALDI-TOF mass spectrometry for protein identification has been used to investigate developing olive fruits. The *cultivar* (*cv.*) Coratina was chosen as reference variety, because of its very high phenolic content. The total protein content extracted from drupe mesocarp at three different developmental stages (45, 110 and 150 DAF -days after flowering) was analysed in order to monitor major proteome changes during fruit development and to reveal modulation in the biosynthesis of compounds related to major quality traits of olives and oil.

## Results

Fruit development is a complex phenomenon unique to plant species, which displays deep changes in biochemistry, physiology, gene and protein expression of the fruit. These changes are a combination of events, which are under strict genetic control and influenced by several environmental conditions, as well. Proteomics offers the possibility to dig deeper into the major changes during fruit development and to classify temporal patterns of protein accumulation occurring during this multifaceted phenomenon. In this work, we started monitoring the proteome variations in order to shed light on the complex metabolic changes underlying fruit development in *Olea europaea*.

### Protein Extraction from Mesocarp and Epicarp of Olive Drupes

**Figure 1:**
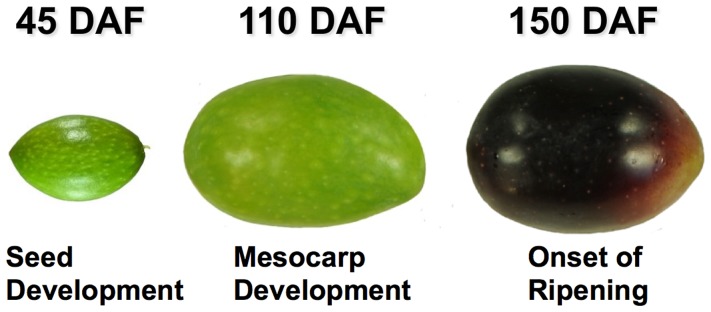
Olive fruit samples. Representative image of the olive drupes harvested at 45, 110 and 150 DAF, used for the comparative proteomic analysis. The stages correspond to important physiological phases of fruit development: II) seed development, IV) mesocarp development and V) onset of ripening, respectively.

**Figure 2:**
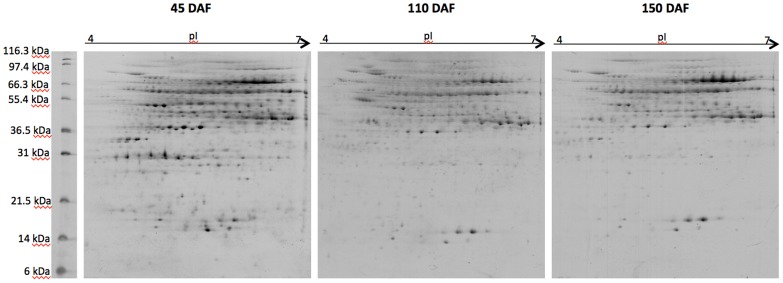
Olive drupe proteome. Typical 2-DE gel patterns in the 4–7 pH range of proteins extracted from drupe at 45, 110 and 150 DAF. 200 ug of proteins were loaded on each gel stained by using SYPRO Ruby (Bio-Rad). The marker is Mark12™ Protein Standard (Invitrogen).

**Figure 3:**
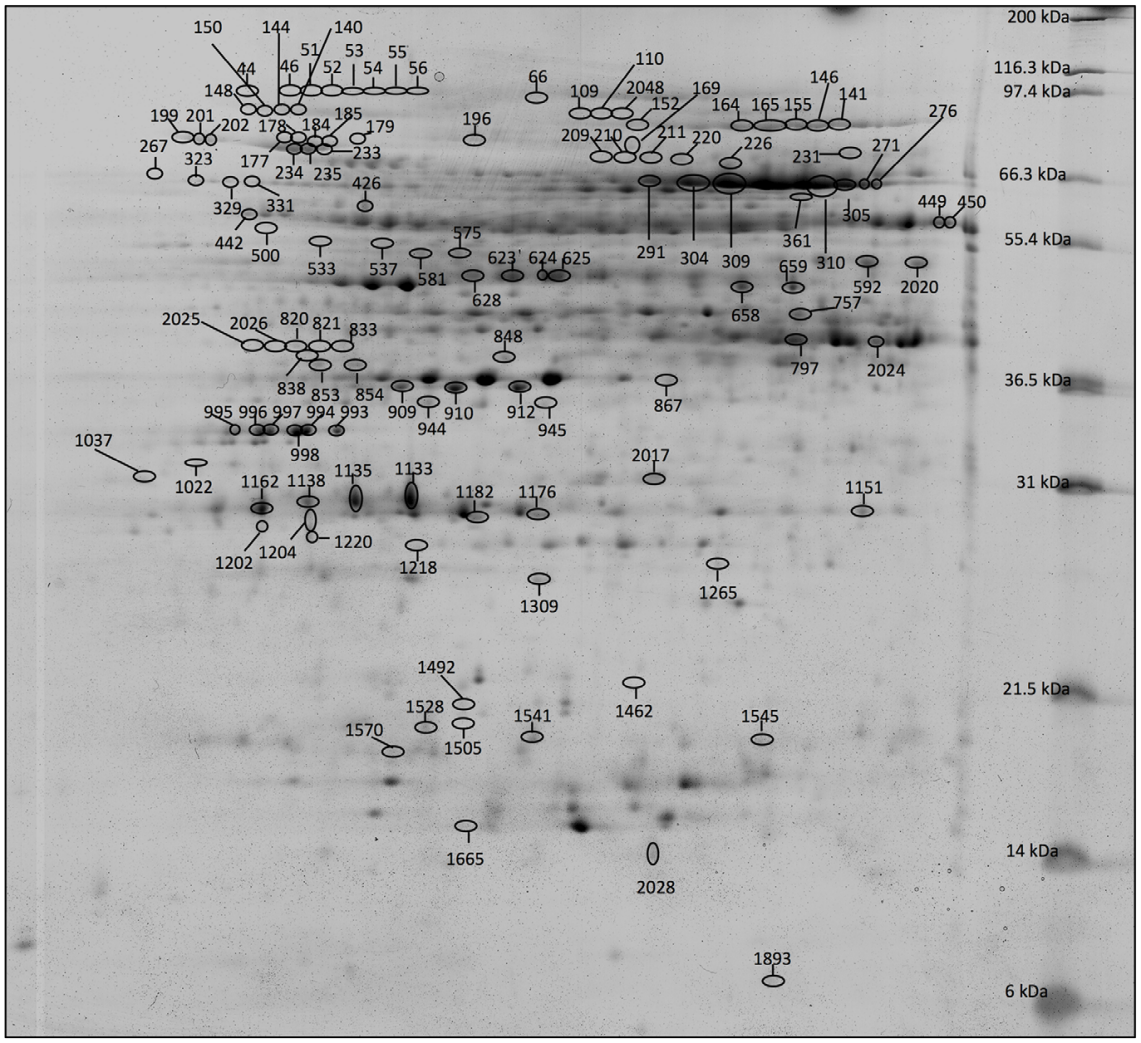
Labeled reference gel. Differentially accumulated protein spots identified by MALDI TOF mass spectrometry are reported as circled spots. The reported spot numbers correspond to those in Table 1.

Total protein content was extracted from olive fruits at three different developmental stages (Figure 1), after pit removal. Extracts were separated on 2-DE gels and stained with Sypro Ruby (Bio-Rad). In Figure 2, the images corresponding to the proteins extracted from olive drupes at 45, 110 and 150 DAF are reported. Approximately, 1,600 protein spots were detected, per developmental stage, during image analysis performed by using Progenesis SameSpots (version 3.3, Nonlinear Dynamics). To our best knowledge, the 2-D protein profile shown here represents the first proteome map of olive fruit (Figure 2 and Figure 3). So far, only a couple of works focused on olive proteome have been reported in literature [9], [10]. These studies were limited by a common major drawback in plant proteomics: the difficulty in obtaining high quality protein extracts. For 2-DE separation and analysis, we have used a classical phenol extraction method [11] with minor modification to remove major contaminants, *i.e.*, phenolics and oil, affecting 2-DE separation (as reported in Materials and Methods).

### Image Analysis and PCA

Proteins extracted from drupes at 45, 110 and 150 DAFs were separated on 2-DE. To ensure statistical reproducibility, four technical replicates were run from each sample, generated from a pool of at least four different olive drupes. Initially, the pH 3–10 IPG linear strips (18 cm; data not shown) were used for separation in the first dimension to get an overview of the olive proteome distribution on the 2-DE. However, since the vast majority of spots clustered at pH 4–7, IEF in pH 4–7 was applied to optimize spot resolution in the densely populated area of the 2-D gel (Figure 2).

**Figure 4:**
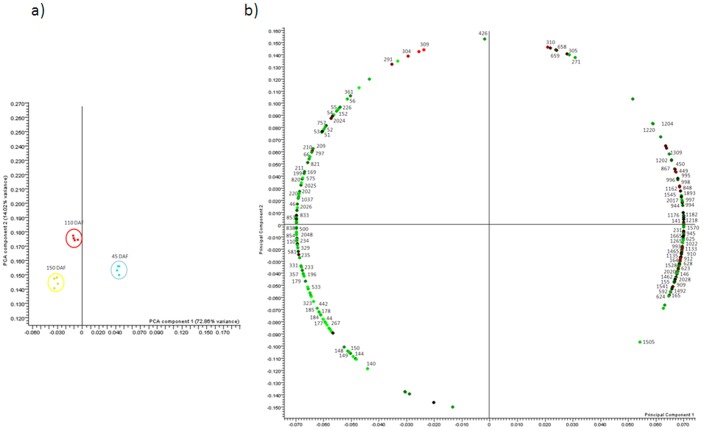
Principal component analysis. Principal component analysis (PCA) of differentially accumulated protein spots. Score plot and loading plot are reported in panel a and panel b, respectively.

Statistical analysis elaborated with Progenesis SameSpots software (Nonlinear Dynamics) revealed 247 protein spots differentially accumulated in the fruit during development. Spot abundance fold change ≥2, ANOVA (p value) ≤0.02 and false discovery rate (q value) ≤0.01 were used to define differentially accumulated protein spots. Most of differentially accumulated protein spots appear as train of spots (shifted in pI), especially at high molecular masses. Principal Component Analysis (PCA) was also performed in order to identify the most relevant features of the data set retrieved from the 2-DE gels (Figure 4). As expected, the samples completely segregate among the developmental stages. The first PCA component explains 72% of the variance, indicating that the stage of development is the largest source of variation. The second PCA component (14% variance), interestingly, separates 110 DAF with respect the other stages. However, our data do not allow an obvious correlation to the involved biological processes (Figure 4).

### Protein Spots Changing in Abundance during Olive Drupe Development

The image analysis revealed 247 protein spots as differentially accumulated. Of them, 170 were manually excised from the gel, digested with trypsin and subjected to mass spectrometry. The remaining 77 differentially accumulated spots, as identified by the image analysis software (Progenesis SameSpot - Nonlinear Dynamics), appeared too faint to be manually picked up and were not considered for further analysis. 121 out of the 170 protein spots were successfully identified (Figure 3). They correspond to 68 unique proteins since several proteins appeared in more than one spot (Table 1, Figure 3). The presence of a same protein in multiple spots can be due to post-translational modifications (PTM) [12], splice variants, protein degradation, or allelic variation [13]. At this stage of the analysis, it is not possible to know whether these multiple forms correspond to products of different yet related genes or to post-translational modifications of the same gene product. Isoelectric heterogeneity in 2-DE is very common in plant proteomics. These putative PTM would not ordinarily have been found using genomic and transcriptomic approaches, thus reinforcing the utility of proteomics to identify these specific changes as likely tuning mechanisms of the biological processes under investigation. The negative outcome for protein identification of the remaining spots (49) can be generally correlated to the lack of known protein and nucleotide sequences for olive tree and for its entire clade. In some cases, the quality of collected mass spectra was low as a consequence of the poor detection of low-abundant protein spots.

**Table 1:** Protein identifications of differentially abundant spots.

Function	Spot number	Fold Change	Experimental pI	Experimental MW (kDa)	Accession/TC identifier	Protein name/blast result	Organism	Combined Score	E-value (Combined Score)	PMF score	E-value (PMF Score)	Seq. Coverage %	PMF peptides	n° of MS/MS sequenced peptides
Allergenes														
	909	7,55	5,27	36198	gi|218963723	Ole e 12.01 allergen	Olea europaea	116	0,0000021			0,19	5	1
	910	7,11	5,63	36198	gi|218963723	Ole e 12.01 allergen	Olea europaea	154	3,4E-10			0,27	8	1
	912	8,06	5,44	36137	gi|218963723	Ole e 12.01 allergen	Olea europaea	192	5.4e-14			0,47	11	1
	867	2,01	6,11	37328	contig01870	Ole e 12.01 allergen	Olea europea	88	0.00099			0,1	4	1
Amino acid metabolism														
	625	2,00	5,75	50709	gi|3024122	S-adenosylmethionine synthase 2	Oryza sativa	152	5.6e-10			0,22	9	2
	628	2,67	5,5	50572	gi|75308025	S-adenosylmethionine synthase 2	Elaeagnus umbellata	164	3.5e-11			0,21	8	2
	592	3,88	6,71	53055	gi|225441193	S-adenosylmethionine synthase 3 isoform 2	Vitis vinifera	174	4.1e-12			0,26	9	2
	623	2,27	5,62	50709	gi|224064730	s-adenosylmethionine synthetase 5	Populus trichocarpa	245	2.8e-19			0,26	8	4
	2020	2,71	6,83	52020	gi|224068797	s-adenosylmethionine synthetase 6	Populus trichocarpa	143	4.4e-09			0,17	7	1
	624	2,36	5,72	50709	contig01699	s-adenosylmethionine synthetase	Olea europea			86	0.0015	0,24	9	
	141	4,98	6,58	89000	gi|296085909	methionine synthase	Vitis vinifera	266	2.1e-21			0,18	11	2
	146	4,52	6,54	88710	OLEEUCl007333:Contig7	methionine synthase	Olea europea	219	7.7e-017			0,18	10	3
	155	3,67	6,51	88421	contig00191	methionine synthase	Olea europea	358	1E-30			0,21	11	3
	164	3,59	6,29	87841	contig00103	methionine synthase	Olea europea			78	0.011	0,13	10	
	165	2,89	6,4	87841	contig00103	methionine synthase	Olea europea	405	2E-35			0,22	16	2
Cell cycle														
	46	3,04	5,06	96821	OLEEUCl010852:Contig3	cell division control protein	Olea europea	113	3.1e-006			0,12	7	3
	51	4,78	5,17	96241	OLEEUCl010852:Contig3	cell division control protein	Olea europea	181	4.9e-013			0,13	10	3
	52	4,64	5,21	96241	OLEEUCl010852:Contig3	cell division control protein	Olea europea	279	7.7e-023			0,28	20	3
	66	2,39	5,59	95662	gi|224140199	cell division cycle protein 48 homolog	Populus trichocarpa	154	3.4e-10			0,16	10	2
Cellular organization, communication and signal transduction														
	1037	2,25	4,59	32632	contig02305	14-3-3 protein	Olea europea			86	0.0017	0,28	8	
	533	5,86	4,97	55400	gi|37936216	Tubulin, alpha-tubulin	Miscanthus floridulus	90	0.00087			0,14	6	1
	537	3,19	5,1	55400	contig01407	Tubulin, alpha-tubulin	Olea europea	94	0.00026			0,1	4	1
	500	3,27	4,87	58586	gi|217071826	tubulin, beta	Medicago truncatula	174	3.5e-12			0,34	13	2
Detoxification/oxidation-reduction process														
	1176	3,20	5,7	29299	contig03404	ascorbate peroxidase	Olea europea			104	2.5e-005	0,27	9	
	1182	2,91	5,51	29204	contig03404	ascorbate peroxidase	Olea europea			127	1.3e-007	0,35	11	
	2017	2,87	6,04	32843	OLEEUCl027825:Contig2	ascorbate peroxidase	Olea europea			105	1.9e-005	0,38	12	
	449	4,12	6,9	61772	gi|20138726	catalase	Suaeda salsa	140	8.8e-09			0,12	7	2
	450	5,41	6,93	61772	gi|1345684	Catalase isozyme 3	Nicotiana plumbaginifolia	119	1.1e-06			0,24	12	2
	944	2,32	5,36	35291	OLEEUCl011602:Contig3	lactoylglutathione lyase	Olea europaea	150	6.1e-010			0,19	6	2
	945	2,42	5,71	35291	contig03001	lactoylglutathione lyase	Olea europaea			72	0.041	0,22	8	
Energy and carbon metabolism														
	271	3,47	6,82	73069	gi|25989474	beta-glucosidase	Olea europaea	82	0.0054			0,13	7	1
	276	2,38	6,76	72779	gi|25989474	beta-glucosidase	Olea europaea	80	0.0096			0,13	7	2
	291	2,28	6,05	72490	gi|25989474	beta-glucosidase	Olea europaea	283	4.3e-23			0,28	14	1
	304	2,24	6,15	72200	gi|25989474	beta-glucosidase	Olea europaea			102	0,000054	0,37	14	
	305	2,55	6,71	72200	gi|25989474	beta-glucosidase	Olea europaea	144	3.4e-09			0,16	8	3
	309	2,00	6,27	71910	gi|25989474	beta-glucosidase	Olea europaea	101	0,00007			0,33	12	1
	310	2,16	6,63	71910	gi|25989474	beta-glucosidase	Olea europaea			84	0,0031	0,27	14	
	109	5,77	5,74	90738	OLEEUCl012853:Contig3	hydrolase, hydrolyzing O-glycosyl compounds	Olea europaea	119	7.7e-007			0,15	8	3
	110	3,31	5,81	90738	OLEEUCl012853:Contig3	hydrolase, hydrolyzing O-glycosyl compounds	Olea europaea	217	1.2e-016			0,16	8	1
	2048	3,74	5,89	90738	contig00575	hydrolase, hydrolyzing O-glycosyl compounds	Olea europea	148	0,000000001			0,09	6	2
	797	2,53	6,49	42432	OLEEUCl022518:Contig2	NAD-dependent glyceraldehyde 3-P dehydrogenase	Olea europea	202	3.9e-015			0,36	11	1
	44	2,85	4,77	97400	OLEEUCl021071:Contig3	Glucose-regulated protein 94 homolog	Olea europea	150	6.1e-010			0,13	5	2
	209	3,25	5,9	81759	contig01717	transketolase	Olea europea	100	7.2e-005			0,12	4	1
	210	2,97	5,97	81759	gi|2501356	Transketolase, chloroplastic	Solanum tuberosum	87	0.0019			0,04	3	1
	169	3,32	6	85814	OLEEUCl058696:Contig1	transketolase	Olea europea	133	3.1e-008			0,13	8	1
	361	2,09	6,48	68724	contig00314	pyrophosphate-dependent phosphofructokinase β-subunit	Olea europea			100	6.4e-005	0,23	16	
	853	3,09	5,09	39535	gi|226529151	pyruvate dehydrogenase E1 component subunit beta	Zea mays	161	7E-11			0,19	8	3
	854	3,29	5,2	39535	gi|226529151	pyruvate dehydrogenase E1 component subunit beta	Vitis vinifera	146	2.6e-09			0,16	7	2
	2024	2,90	6,77	42363	contig03754	Glyceraldehyde-3-phosphate dehydrogenase	Olea europea			75	0.025	0,25	6	
Lipid synthesis														
	820	7,01	4,95	40915	gi|25989478	enoyl ACP reductase	Olea europaea	132	0,000000054			0,15	7	3
	821	5,17	5,09	40915	gi|25989478	enoyl ACP reductase	Olea europaea	152	5,4E-10			0,13	6	1
	833	2,36	5,13	40087	gi|25989478	enoyl ACP reductase	Olea europaea	97	0.00018			0,12	5	3
	838	3,18	4,98	40018	gi|25989478	enoyl ACP reductase	Olea europaea	71	0,004861111			0,1	5	2
	2025	4,38	4,8	42984	gi|25989478	enoyl ACP reductase	Olea europea	272	5.6e-22			0,13	6	1
	2026	6,39	4,84	43053	gi|25989478	enoyl ACP reductase	Olea europea	329	1.1e-27			0,27	11	1
	152	2,34	5,95	88421	contig02468	acetyl co-enzyme A carboxylase carboxyltransferase α-subunit	Olea europea	1776	1.6e-012			0,13	5	2
	53	4,72	5,23	96241	gi|187960379	lipoxygenase 2	Olea europaea	107	1.7e-05			0,1	10	2
	54	3,95	5,26	96241	gi|187960379	lipoxygenase 2	Olea europaea	151	6.8e-10			0,14	14	1
	55	4,18	5,28	96241	gi|187960379	lipoxygenase 2	Olea europaea	104	3.4e-05			0,17	14	3
	56	3,63	5,33	96241	gi|187960379	lipoxygenase 2	Olea europaea	176	2.1e-12			0,14	13	1
Malate metabolic process														
	211	2,82	6,05	81759	contig00590	NADP-malic enzyme	Olea europea	227	1.3e-017			0,23	14	3
	220	2,61	6,16	81179	contig00590	NADP-malic enzyme	Olea europea			83	0.0032	0,17	10	
	226	2,21	6,28	80890	OLEEUCl012956:Contig1	NADP-malic enzyme	Olea europea	201	4.9e-015			0,15	8	3
Miscellaneous														
	1462	5,16	5,98	21595	gi|20339439	maturase K	Chelone lyonii			74	0.037	0,09	5	
	1135	4,46	5,13	29818	contig03639	acetone-cyanohydrin lyase	Olea europea			84	0.0027	0,28	9	
	1133	4,97	5,3	29960		acetone-cyanohydrin lyase	Olea europea	260	6.4e-021			0,3	9	2
	331	3,83	4,73	70752	gi|3928134	annexin P38	Capsicum annuum			78	0.015	0,19	7	
	848	2,03	5,75	39811	OLEEUCl014405:Contig1	annexin	Olea europeae			121	4.9e-007	0,27	11	
	658	2,02	6,32	49744	contig01144	GDP-D-mannose 3',5'-epimerase	Olea europea			95	0,00022	0,2	9	
	659	2,02	6,48	49744	gi|319739579	GDP-mannose-3,5-epimerase	Citrus unshiu			81	0.0065	0,31	10	
	267	11,04	4,49	73503	OLEEUCl011314:Contig1	NO BLAST	Olea europea			174	2.4e-012	0,29	7	
	2028	3,13	6,08	14820	EX896161	Unknown	Raphanus sativus			99	0.017	0,33	8	
	1665	2,27	5,47	15912	gi|242069499	hypothetical protein SORBIDRAFT_05g027220	Sorghum bicolor			74	0.031	0,18	11	
	231	2,05	6,73	80455	F7KHMQ104I8XHN	NO BLAST	Olea europea			77	0.014	0,59	6	
	1570	2,38	5,25	18769	OLEEUCl031202:Contig1	NO BLAST	Olea europea			70	0.066	0,19	5	
	757	2,13	6,6	45053	gi|158564568	PII protein	Paeonia suffruticosa			74	0.033	0,53	10	
	1204	4,01	5	28542	gi|326516492	predicted protein	Hordeum vulgare			73	0.043	0,23	7	
	1151	2,23	6,7	29677	gi|168002144	Predicted protein	Physcomitrella patens subsp. patens	114	3.5e-06			0,24	6	1
	1465	2,31	5,47	21500	gi|168045250	predicted protein	Physcomitrella patens subsp. patens			74	0.034	0,2	7	
	1265	3,01	6,26	27172	BG448091	quinone reductase family protein	Medicago truncatula	108	0.0021			0,18	3	2
	323	3,37	4,58	71041	contig02104	protein disulfide isomerase	Olea europea	98	0.00011			0,17	5	1
	1022	2,55	4,73	32874	gi|225440205	thioredoxin-related protein isoform 2	Vitis vinifera			73	0.044	0,23	6	
	1545	2,23	6,39	19399	F7KHMQ103GU2HK	Phosphatidylinositol-4-phosphate 5-kinase	Olea europea			73	0.034	0,51	5	
Phenylpropanoid metabolism														
	1492	3,87	5,47	20744	gi|162949342	4-coumarate:coenzyme A ligase 1	Physcomitrella patens subsp. patens			79	0,000694444	0,09	6	
Photosynthesis														
	1202	2,55	4,85	28542	OLEEUCl010166:Contig3	chlorophyll A/B binding protein	Olea europea	161	4.9e-011			0,13	5	1
	1220	4,81	5	28235	OLEEUCl010166:Contig3	chlorophyll A/B binding protein	Olea europea	200	6.1e-015			0,13	5	2
	1162	3,76	4,85	29440	gi|28630973	chlorophyll a/b-binding protein precursor	Citrus limon	169	1.1e-11			0,14	4	2
	994	3,28	4,98	33538	OLEEUCl010749:Contig3	oxygen evolving complex 33 kDa photosystem II protein	Olea europea	219	7.7e-017			0,39	15	2
	995	2,86	4,77	33478	OLEEUCl010749:Contig3	oxygen evolving complex 33 kDa photosystem II protein	Olea europea			111	4.9e-006	0,29	10	
	996	2,88	4,85	33478	OLEEUCl010749:Contig3	oxygen evolving complex 33 kDa photosystem II protein	Olea europea	145	1.9e-009			0,31	10	1
	997	3,92	4,87	33478	contig02647	oxygen evolving complex 33 kDa photosystem II protein	Olea europea	86	0,0015			0,16	4	1
	998	2,69	4,96	33478	OLEEUCl010749:Contig3	oxygen evolving complex 33 kDa photosystem II protein	Olea europea	142	3.9e-009			0,25	9	1
	993	2,31	5,08	33599	gi|326467059	oxygen evolving enhancer protein 1	Litchi chinensis			74	0.031	0,21	6	
	1309	3,01	5,7	26368	OLEEUCl011082:Contig2	Oxygen-evolving enhancer protein 2	Olea europea			72	0.036	0,23	5	
	1893	2,80	6,43	7000	F7KHMQ104INWLL	photosystem I iron-sulfur left	Olea europea	220	6.4e-017			0,29	4	3
	329	3,06	4,69	70752	gi|1351030	RuBisCO large subunit-binding protein subunit alpha	Brassica napus			73	0.049	0,17	8	
Protein synthesis/storage														
	1218	2,09	5,27	28353	OLEEUCl048557:Contig1	eIF3 - Eukaryotic translation initiation factor 3 subunit	Olea europea	172	3.9e-012			0,15	4	2
	581	2,07	5,23	53469	contig00916	eIF-4A - dead box ATP-dependent RNA helicase	Olea europea			74	0.026	0,21	8	
	575	4,37	5,42	53607	gi|222424799	eIF-4A -dead box ATP-dependent RNA helicasee	Arabidopsis thaliana			77	0.017	0,29	8	
Stress response/Protein folding														
	199	2,76	4,62	82628	contig00166	heat shock 70 kDa	Olea europea	297	1.3e-024			0,13	8	4
	201	3,47	4,65	82338	contig00166	heat shock 70 kDa	Olea europea	87	0.0012			0,11	4	1
	202	3,12	4,68	82338	contig00166	heat shock 70 kDa	Olea europea	137	1.3e-008			0,12	8	1
	179	3,97	5,04	84076	gi|45331281	heat shock 70 kDa cognate protein 1	Vigna radiata	251	6.8e-20			0,28	16	3
	235	2,54	4,98	80021	contig05105	heat shock 70 kDa cognate protein 1	Olea europea	243	3.2e-019			0,46	11	2
	233	3,23	5,03	80310	gi|15241849	heat shock 70kDa protein 1/8	Arabidopsis thaliana			150	0,000000001	0,36	14	
	178	9,41	4,93	84076	contig00108	heat shock protein 70 kDa	Olea europea	201	5.1e-015			0,14	11	2
	234	2,63	4,94	80021	OLEEUCl003515:Contig10	heat shock protein 70 kDa	Olea europea	162	3.9e-011			0,23	13	1
	177	7,49	4,88	84076	contig00108	heat shock protein 70kDa	Olea europea	205	2E-15			0,13	11	2
	140	5,74	4,88	89000	contig00172	heat shock protein 90-2	Olea europea	318	1E-26			0,26	20	2
	144	8,83	4,86	88710	contig00159	heat shock protein 90-2	Olea europea	296	1.6e-024			0,27	17	3
	149	5,88	4,82	88421	OLEEUCl011019:Contig3	heat shock protein Hsp90-1	Olea europea	110	6.1e-006			0,2	5	2
	148	4,40	4,8	88421	gi|38154482	heat shock protein Hsp90-1 molecular chaperone	Nicotiana benthamiana	85	0.0025			0,08	4	1
	150	6,77	4,84	88421	gi|38154485	Heat shock protein Hsp90-2 molecular chaperone	Nicotiana benthamiana	304	3.4e-25			0,23	18	3
	185	7,34	4,99	83786	gi|255555659	heat shock protein	Ricinus communis	217	1.7e-16			0,25	17	1
	184	7,61	4,95	83786	OLEEUCl009415:Contig2	heat shock protein	Olea europea	226	1.5e-017			0,2	13	2
	196	2,31	5,65	83207	gi|123656	heat shock 70 kDa	Spinacia oleracea	119	1.1e-06			0,2	8	3
	1528	3,85	5,36	19820	FR642751	small heat shock protein	Fraxinus excelsior	104	0.0054			0,15	3	1
	1541	9,99	5,69	19441	gi|307837689	small heat shock protein	Olea europea	135	2.8e-08			0,24	4	1
	1505	2,88	5,47	20408	contig05929	small molecular heat shock protein	Olea europea	112	0,000004			0,12	3	2
Transport														
	442	2,82	4,75	61772	gi|15233891	V-ATPase B subunit	Arabidopsis thaliana	117	1.8e-06			0,07	4	2
	426	2,20	5,16	64959	gi|283794953	ATPase alpha subunit	Olea europaea	502	5.4e-45			0,39	23	2

For protein identification by peptide mass fingerprinting (PMF), a significant Mascot score (P≤0.05) and at least 5 matched peptides were required. Identifications based on three matched peptides were accepted if significant scores were obtained for at least one peptide by fragment ion mapping. For the identification by combining PMF and fragment ion mapping, the combined score (Combined Score; assigned by Mascot) has been reported.

The identified proteins belonged to a diverse set of pathways and processes (Table 1). Seventeen different protein spots corresponding to 70 kDa and 90 kDa heat shock proteins (HSP) were identified as strongly increasing in abundance during olive drupe development (Table 1, Table S2).

Among proteins accumulating during fruit development, we identified several isoforms of cell division control protein, 3 tubulins, 3 hydrolases, 3 transketolases, 2 beta-subunits of pyruvate dehydrogenase E1 complex, a protein disulfide isomerase and a 14-3-3 protein (Table 1, Table S2). An interesting accumulation trend was also observed for proteins such as enoyl ACP reductase, lipoxygenase 2 and NADP-malic enzyme (Table 1, Table S2).

**Figure 5:**
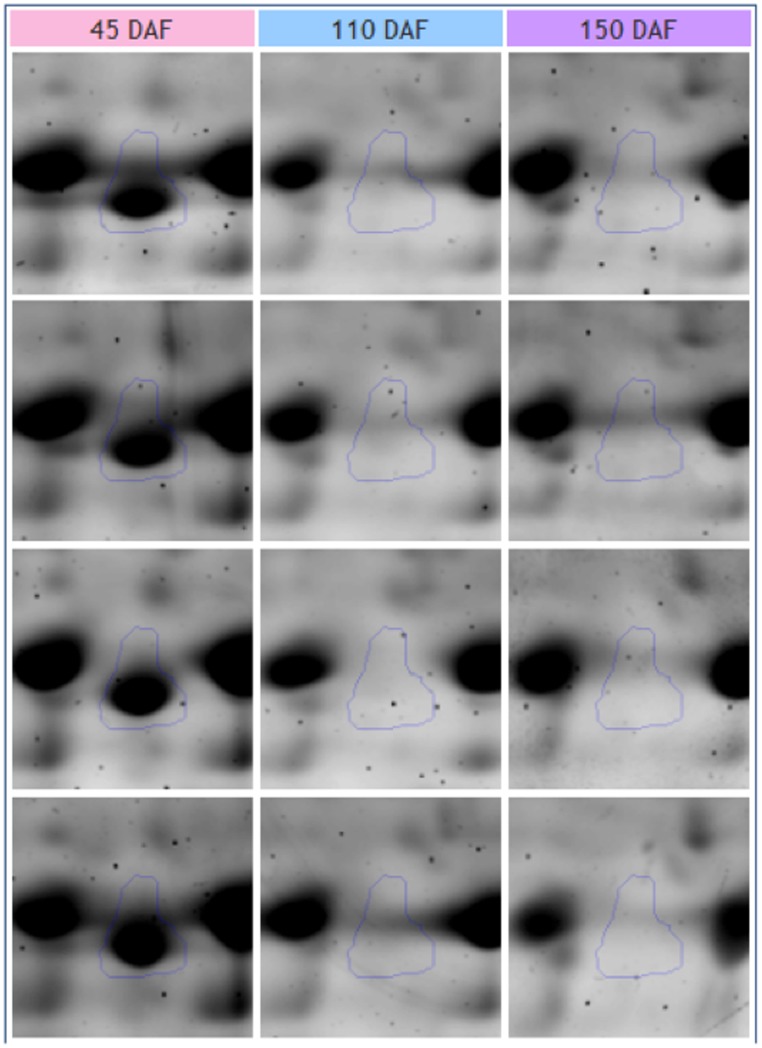
Detail of the 2-DE Analysis. Accumulation pattern of Ole-e-12.01 allergen during olive drupe development elaborated with Progenesis SameSpot (Nonlinear Dynamics).

Reversely, a sharp reduction was detected for protein spots related to Ole-e-12.01 allergen (Figure 5) and to several isoforms of methionine synthase, S-adenosylmethionine synthase, ascorbate peroxidase, lactoylglutathionelyase, catalase, chlorophyll A/B binding protein and for different proteins related to oxygen evolving complex (Table 1, Table S2). Among proteins decreasing, we found small HSPs, acetone-cyanohydrin lyase, thioredoxin-related protein isoform 2, phosphatidylinositol-4-phosphate 5-kinase and 4-coumarate: coenzyme A ligase 1. Finally, several isoforms of β-glucosidase were also identified. They showed a distinctive accumulation pattern, with a peak of accumulation at 110 DAF (Table 1, Table S2).

### Functional Characterization of Differentially Accumulated Proteins

**Figure 6:**
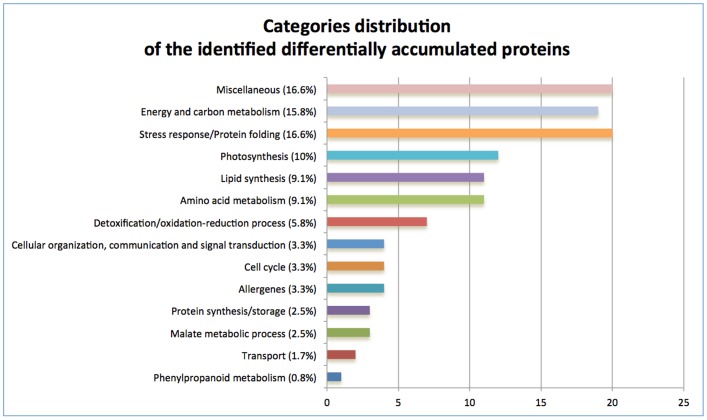
Functional distribution of identified proteins. The proteins showing a statistically significant change were manually sorted into 15 functional categories. The number of hits that match each functional category in reported on x-axis.

In order to generate an overview of the most relevant biological processes involved in olive drupe development, differentially accumulated proteins were individually classified by their putative function on the basis of data available in literature and/or using the information available in the Expasy portal (www.expasy.org). As expected, proteins associated with energy, carbon metabolism and photosynthesis represented the major functional groups showing changes (15.8%) (Figure 6). Several other differentially accumulated proteins are involved in stress responses, lipid and aminoacid metabolism. Moreover, a considerable number of proteins with heterogeneous functions was classified as Miscellaneous (16.6%). This group also includes proteins with not yet identified function, 3 of which (spots 267, 231 and 1570; Table 1, Table S1) did not show homology to any known protein and therefore could be considered specific proteins of the olive species.

### Comparison between Transcript and Protein Abundances

Proteins identified as differentially accumulated during olive fruit development were compared to their putative transcripts, in order to evaluate if changes observed at protein level were consistent with changes at mRNA level. To reach this goal, proteomic data produced in this work were compared with transcriptomic data elaborated during a foregoing study conducted in our laboratories [6] (http://454reads.oleadb.it), where transcripts from *Coratina* and *Tendellone* genotypes at 45 and 135 DAF were analyzed by comparative 454 pyrosequencing.

Since many protein spots identified in this study correspond to different or post-translationally modified forms and/or to different sub-units of the same protein (Table 1), they have been organized into groups according to their putative function (Table S3). Each protein group was then compared to their putative encoding transcripts, eventually traceable in the list of TCs provided as supplementary information in our previous work [6]; (http://www.biomedcentral.com/content/supplementary/1471-2164-10-399-S1.xls).

Comparison between proteomic and transcriptomic datasets revealed that most of the proteins and their putative transcripts showed a similar pattern during drupe development.

With the only exception of RuBisCO large subunit-binding protein subunit alpha, proteins and transcripts related to photosynthesis (chlorophyll A/B binding protein, oxygen evolving complex 33 kDa photosystem II protein, oxygen-evolving enhancer protein) showed a gradual decrease during olive drupe development. By contrast, proteins and transcripts associated with fatty acids biosynthesis and metabolism (enoyl ACP reductase and lipoxygenase) and with heat shock family, strongly increased in abundance. Furthermore, with the exception of beta tubulin (spot 500, Table 1, Table S3) proteins as well as transcripts linked to cell cycle increased in abundance during olive drupe development.

Transcript and protein profile comparison also revealed some divergent patterns, indicative of possible post-transcriptional events. Transcripts corresponding to ascorbate peroxidase, catalase, thioredoxin-related protein and eIF3 subunit, were found to be accumulated during development, whereas the putative corresponding protein spots showed a progressive decrease in abundance (Table S3). An opposite situation has been observed for eIF4 protein spots (Table S3). In this case, the lower abundance of the corresponding transcript might reflect a more rapid transcript turnover.

## Discussion

### Proteins Related to the Developmental Processes

The genotype investigated in this study was the *cv.* Coratina. Fruits were harvested at 45, 110 and 150 DAF, corresponding to the II, IV and V developing phases, respectively. The phase II is characterized by a period of rapid fruit growth, due to both, development of endocarp and intense cell division, the phase IV is marked by a mesocarp development, mainly due to the expansion of pre-existing flesh cells, whereas in phase V oil accumulation reaches the completion [2]. Proteins were extracted from the collected drupe samples, after pit removal. Therefore, the proteomic analyses reflect the changes of protein accumulation in olive mesocarp and epicarp during fruit development.

Our proteomic investigation revealed four differentially accumulated protein spots strictly related to cell division. Spots 46, 51 and 52 (Table 1) correspond to cell division control proteins, while spot 66 (Table 1) was identified as cell division cycle protein 48 homolog. All these protein spots increased in abundance from 45 to 110 DAF (Table S2), and remained approximately stable during the transition from 110 to 150 DAF (Table S2). These results are consistent with the fact that in olive fruits after the pit hardening phase cell division almost ceases while the rate of cell expansion increases [2].

Cell expansion requires cell wall elongation and accumulation of solutes within the vacuole. The vacuolar H+-ATPase (V-ATPase) is a multi-subunit enzyme, which generates a proton electrochemical gradient across the vacuolar membrane. In mature cells, the vacuoles generate and maintain turgor pressure, the driving force for cell expansion. V-ATPase expression is relatively high all over the growth of many fruits, such as peach [14] tomato [15] and cherry tomato [16] suggesting a central role during fruit development. As a matter of fact, Amemiya and colleagues [17] demonstrated that fruit specific V-ATPase suppression reduces fruit growth and seed formation in antisense-transgenic tomato. We identified the B subunit of V-ATPase (spot 442;Table 1) as differentially accumulated protein spot during fruit development; in particular, the correspondent protein spot accumulated throughout olive drupe growth (Table S2), in accordance with previous proteomics studies conducted in tomato and papaya fruits [16], [18].

Annexins are a large family of ubiquitous, calcium- and membrane-binding proteins. They are potentially involved in cell expansion, due to their function in Golgi-mediated secretion of polysaccharide precursors for cell wall synthesis [19], [20]. Annexin expression is elevated during fruit development, when massive structural remodelling of the cell wall takes place [21], [22]. In tobacco, annexin P34 is supposed to participate in the vacuolation process of expanding cells [23]. In strawberry and pepper, gene expression analyses reported an increment of annexin during development until fruit ripening [21], [22]. It has been speculated that annexin-like proteins might influence ion fluxes, membrane cytoskeletal attachments, or other aspects of plasmalemma function that change during fruit maturation and senescence [21]. Interestingly, two protein spots (spots 331 and 848; Table 1), corresponding to two different proteins belonging to annexin family, were identified in this work. These two protein spots showed an opposite trend of accumulation during fruit development (Table S2). Annexin P38 (spot 331; Table 1; Table S2) increased progressively throughout the investigated time points, while the putative annexin protein identified in spot 848 (Table 1; Table S2) decreased. A similar contrasting trend was already observed in our previous proteome survey of strawberry fruits during ripening [24], reinforcing the clues for a role of annexins in fruit ripening. It should be taken into account that annexins are a large family of multifunctional proteins; hence it is plausible that their expression pattern can be independently regulated.

The changes undergone by cells, which first divide and then expand, must be supported by changes in cytoskeleton structure. A microtubule-associated protein homolog and a tubulin homolog were up-regulated during watermelon fruit development [25]. Two alpha- and one beta-tubulin protein spots were found to be accumulated also in olive fruits: the corresponding spot volumes (Table S2) regularly increased throughout drupe development, supporting the role of tubulin subunits in the whole process of cell size enlargement.

The developmental process is also influenced by the type of the fruit, either climacteric or non-climacteric. In climacteric fruits, the ripening phase is characterized by a peak in respiration and a burst of ethylene, which accompanies changes in color, texture, flavor and aroma; on the other hand, non-climacteric fruits show no dramatic changes in respiration and ethylene production remains at very low level, although similar major visual, texture, flavor and metabolic changes occur as in climacteric fruits. It has been suggested that both ethylene-dependent and ethylene independent mechanisms can coexist to co-ordinate the dramatic changes occurring during ripening [26]. Many of these events have been investigated and characterized in climacteric-ripening fruits, whereas non-climacteric ripening is still poorly understood. Olives are classified as non-climacteric fruits and the ethylene production has been reported to be non-detectable [27]. Notwithstanding, olive drupes can produce ethylene and respond to ethylene after application of 1-aminocyclopropane-1-carboxylic acid (ACC) to the surface of the fruit, suggesting a presumptive block in the pathways producing ethylene [28]. Besides its importance in protein synthesis, methionine is supposed to play a central role as precursor of ethylene. In tomato, it has been demonstrated that methionine is a rate limiting metabolite for the ethylene synthesis, raising the hypothesis that methionine is part of the mechanism that supports the climacteric ethylene production in tomato fruit [29]. A more recent work on climacteric papaya revealed an increase of S-adenosylmethionine synthetase and methionine synthase during fruit ripening, suggesting they are required for the climacteric burst of ethylene, as proposed for tomatoes [30]. Interestingly, we identified several protein spots associated to methionine synthase (spots 141, 146, 155, 164, 165; Table 1) and S-adenosylmethionine synthetase (spots 625, 628, 592, 623, 2020, 624; Table 1). Their abundance intriguingly decreased during olive drupe development (Table S2), showing an opposite trend with respect to tomato and papaya fruits [29], [30]. This divergent accumulation might be related to the non-climacteric ripening process marking olive fruits. Both enzymes, in fact, belong to the ethylene biosynthetic pathway, where changes in the availability of soluble methionine limit ethylene production [29]. In this context, the decay of methionine synthase and S-adenosylmethionine synthetase in olives could contribute to shed a new light on the poorly understood mechanism of non-climacteric fruit ripening and therefore it deserves further investigations. In addition to their involvement in the ethylene biosynthesis, it should be remarked that methionine synthase and S-adenosylmethionine synthetase are also involved in the biosynthesis of polyamines, which in turn are required for cell growth and cell division [31], [32]. S-adenosyl-L-methionine is, in fact, a common substrate for the biosynthesis of polyamines and the hormone ethylene. In olive, polyamines are putatively involved in cell division and in the developmental acquisition of cell size [33]. In this context, the decreasing levels of methionine synthase and S-adenosylmethioninesynthetase enzymes, revealed in our proteomic investigation, is not surprising. Indeed, we observe high levels of both enzymes at 45 DAF, when cell division occurs at higher rate; while at later stages their ongoing decrease could be driven by both the cessation of cell division and the non-climacteric nature of olive drupe ripening.

### Proteins Related to Fruit Photosynthesis

Developing olives show photosynthetic activity [34], indeed, drupes remain green for a considerable period, retaining active chloroplasts. Chlorophyll A and B and the chlorophyll A-B binding protein compose the light-harvesting complex (LHC), which functions as a light receptor, capturing and delivering excitation energy to photosystems I/II. Spots associated to chlorophyll A-B binding protein (spots 1162, 1202 and 1220; Table 1) were detected as differentially accumulated during olive drupe development. The corresponding spot intensity remained nearly stable when comparing 45 and 110 DAF, while it decreasedat 150 DAF (Table S2).The oxygen-evolving complex (OEC), also known as the water-splitting complex, is a water-oxidizing enzyme involved in the photo-oxidation of water during the light reactions of photosynthesis. Several isoforms of OE33 (spots 994, 995, 996, 997, 998; Table 1) were detected in our study. Moreover, two spots corresponding to the oxygen evolving enhancer protein 1 and 2 (spots 993 and 1309;Table 1) and photosystem I iron-sulfur center (spot 1893; Table 1), were further identified. Densitometric analysis for all these protein spots indicated that the level of proteins belonging to photosynthetic apparatus accumulated in young green olives and decreased during mesocarp development.

These results should be interpreted in the light of the expected progressive degradation of chlorophyll and photosynthetic apparatus associated with transition of chloroplasts to chromoplasts [35]. The gradual decrease of photosynthesis-related protein abundance is an established evidence [16], [36]. In contrast with these observations, throughout olive fruit development, RuBisCO large subunit-binding protein subunit alpha (spot 329; Table 1, Table S2) showed an increase in spot intensity. This protein that belongs to the chaperonin family binds RuBisCO small and large subunits and is implicated in the assembly of the enzyme oligomer. This unexpected accumulation could be the result of regulation mechanisms independent from the plastid differentiation processes.

As proposed by Blanke and Lenz [37], fruit photosynthesis displays characteristics different from either C3 or C4/CAM plants. During fruit developmental period, in fact, cell division and growth are supported by mitochondrial respiration of sugars imported from the phloem. Due to the impermeability of fruit cuticle, CO_2_ produced after this intense metabolism accumulates in high concentrations in the fruit cell-free space. As reviewed by Sanchez and Harwood [38], inorganic carbon is fixed into oxalacetate, converted into malate by malate-dehydrogenase. Malate can be decarboxylated by cytosolic or mitochondrial malic enzyme to yield pyruvate and CO_2_. The latter can further be photosynthetically fixed into triose phosphate in the fruit chloroplasts. It has been demonstrated that fruit photosynthesis contributes to the carbon economy of developing fruits and hence to olive oil biogenesis [38], [39]. Indeed, fruits grown in autotrophic conditions on defoliated branches proportionally accumulate the same oil amount of control fruits, while the oil content of olives grown in the dark in heterotrophic conditions is significantly lower [38], [39]. Interestingly, we identified the NAD-malic enzyme (ME) involved in C4 photosynthesis. Three different putative isoforms of this enzyme (spots 211, 220, 226; Table 1) were detected in our proteomic investigation. The intensity of the related protein spot volume increased in the developmental phase, during the transition from 45 to 110 DAF, while the abundance remained approximately stable during the transition from 110 to 150 DAF (Table S2). On the one hand, the accumulation of ME might represent the proof that in *Olea,* during fruit photosynthesis, re-fixing of CO_2_ occurs. On the other hand, this might explain the contribution of fruit photosynthesis toward the biogenesis of olive oil, as well. As a matter of fact, the reaction catalyzed by ME yields pyruvate, which is the precursor of fatty acid biosynthesis. The malic enzyme accumulates during developmental stages, where an intense oil accumulation is expected to occur [2], suggesting a pivotal role for this enzyme in oleogenesis. In this context, the discussed increase of RuBisCO large subunit-binding protein subunit alpha might find a new possible explanation: this protein could work as chaperonin, stabilizing RuBisCO and thus its activity in fixing CO_2_, yielded by the malic enzyme beside pyruvate production.

### Proteins Related to Fatty Acids Metabolism

Pyruvate is required for the biosynthesis of acetyl-CoA, which is the substrate for synthesis of the carbon backbone of all fatty acids (FA). The oxidative conversion of pyruvate into acetyl-CoA is catalyzed by the pyruvate dehydrogenase complex (PDC). Aceytl-CoA is then irreversibly converted into malonyl-CoA by aceyl-CoA carboxylase (ACC). The condensation between malonyl-ACP generated from malonyl-CoA and aceyl-CoA initiates fatty acids biosynthesis. The elongation of the acyl chain proceeds through several cycles of reduction-dehydration-reduction catalyzed by enzymes belonging to FAS (fatty acid synthases) [2]. In this respect, it is worthy to mention the identification of protein subunits of both PDC and ACC protein complexes. Two protein spots associated with pyruvate dehydrogenase E1 component subunit beta (spots 853 and 854; Table 1) and one protein spot related to acetyl co-enzyme A carboxylase carboxyltransferase alpha subunit (spot 152; Table 1), have been detected in our analysis. Pyruvate dehydrogenase E1 component subunit beta increased in abundance throughout the development (Table S2). Consistent with the accumulation pattern of oil, the carboxyltransferaseacetyl co-enzyme A carboxylase alpha subunit showed a peak of accumulation at 110 DAF, where an intense oil storing is expected [2]. A similar trend in terms of specific protein spot accumulation was also recorded for enoyl-ACP reductase, belonging to FAS enzymes. Six different protein spots (spots 820, 821, 833, 838, 2025, 2026; Table 1) were detected for this protein, which increased during the oleogenic period, with a little or no increase during from 110 DAF to 150 DAF (Table S2). These results seem to be in fairly good accordance with previous genomic studies on olive drupes [7].

The oil accumulated in the drupe mesocarp shows a characteristic acyl composition. The most abundant component is the monounsaturated fatty acid oleate (C18∶1), accounting up to 80% of the total lipidic composition. Other major fatty acids are the saturated palmitic acid (10–20%) and the di-unsaturated linoleic acid (2.5–20%). The balance among these fatty acids plays a significant role on olive oil nutritional properties. The formation of monounsaturated and polyunsaturated fatty acids takes place by desaturation reactions. Strikingly, no fatty acid desaturases or related enzymes were detected in our investigation. Most of the plant fatty acid desaturases can be classified as membrane-bound desaturases or integral membrane proteins [40]. Analysis of membrane proteins is usually more difficult than soluble proteins. Typically, membrane proteins are very strongly under-represented on 2D gels because they tend to precipitate in the IEF gel at their pI and cannot be transferred to the second dimension [41], [42].

### Proteins Associated to Metabolism of Phenolics and Aroma Compounds

Extra virgin olive oil, deriving from mechanical extraction from the fruits, contains high levels of phenolics and volatile compounds deriving from drupes.

The drupes of *cv.* Coratina, are characterized by a very high phenolic content [4], where secoiridoids represent the most important class. They include simple phenols, like tyrosol and hydroxytyrosol, and quantitatively more important conjugated forms like oleuropein, demethyloleuropein and ligstroside. Oleuropein is the main secoiridoid, representing up to 82% of the total bio-phenols and is responsible for the characteristic bitter and pungent taste of the olive drupes and oil. Oleuropein biosynthetic pathway is complex and not yet well understood. Many enzymes and related genes involved in oleuropein metabolism are still unknown [6]. Oleuropein is very abundant at early fruit developemental stages, progressively declining at later phases [43], [44]. Generally, this decrease inversely correlates with the appearance of oleuropein derivatives. Data available in literature suggest an involvement of beta-glucosidase enzymes in oleuropein metabolism, in both anabolic and catabolic routes [45], [46], [47]. Interestingly, seven protein spots (spots 271, 276, 291, 304, 305, 309 and 310; Table 1), corresponding to β-glucosidase enzymes, were identified in this work. All these protein spots showed a similar trend during fruit development, with an accumulation at 110 DAF (Table S2). This result seems to be in accordance with the fate of oleuropein decrease during fruit development, suggesting a likely function of this enzyme in oleuropein metabolism. However, as β-glucosidase enzymes are involved in a myriad of processes, further studies are required to prove the effective involvement of the identified protein spots in the oleuropein metabolism.

The compounds responsible for the aroma of olive oil, such as the so-called “Green Odour Notes”, represent an interesting group, important from both a quantitative and a qualitative point of view. Indeed, the aroma of olive oil, is dominated by the green-smelling odorants Z-3-hexenal, E-2-hexenal, Z-3-hexenyl acetate and Z-3-hexenol [48]. These compounds, characterizing the aroma of freshly cut grass, green apples and foliage, have their origin in the lipoxygenase (LOX) pathway, which requires C18-polyunsaturated fatty acids, such as linoleic and alpha-linolenic acids [49], [50]. The LOX pathway, leading to green notes substances is very well characterized in plants [51] and includes sequential reactions catalyzed by lipoxygenase, hydroperoxide lyase and alcohol dehydrogenase.

It should be noted that our proteomic investigation revealed a number of protein spots associated to LOX2 (spots 53, 54, 55, 56; Table 1). Type 2 LOXs are plastidial proteins producing almost exclusively 13-hydroperoxide derivatives from polyunsaturated fatty acids. Several isoforms of this enzyme increased in abundance from 45 to 110 DAF, while their abundance remained approximately stable during the transition from 110 to 150 DAF (Table 1, Table S2).This result suggests a central role of type 2 LOXs in the generation of fruit aroma compounds, which can be transferred to the oil.

### Comparison between Transcripts and Protein Abundance

To investigate whether changes observed at protein level correspond to variations at the mRNA level, a comparison between protein and transcript profiles was performed. An overall concordance of protein and transcript levels has been here revealed, even if some divergent patterns were also found. To explain both convergent and divergent patterns, it should be taken into account that proteomic comparisons were based on three developmental stages, i.e. 45, 110 and 150 DAF, while transcriptomic comparative studies were performed on two developmental stages, 45 and 135 DAF [6]. Besides, biologically, some divergences between proteins and transcripts are likely to be explained by molecular events such as translational efficiency, alternative splicing, folding, assembly into complexes, transport and localization, covalent modification, secretion, and degradation, all of which affect protein levels independently of transcripts.

In this respect, it is worthy to mention the identification of RuBisCO large subunit-binding protein subunit alpha. The corresponding protein spot increased in abundance during fruit development, while the amount of its putative transcript decreases (Table S2; Table S3). As discussed above, photosynthetic apparatus is expected to be progressively disassembled during fruit development. For RuBisCO large subunit-binding protein subunit alpha, we hypothesized a role as chaperonin, stabilizing RuBisCO and thus its activity in CO_2_ re-fixing. Interestingly, the divergent level of mRNA might suggest the presence of a fine-tuning regulation controlling, at the post-transcriptional level, the turnover of the protein and its accumulation. Indeed, post-transcriptional control can optimize protein abundance and/or enzyme activity, reducing the energetic cost of re-synthesis [52].

Moreover, it should be noted that also proteins and transcripts associated with detoxification and oxidation-reduction processes, in particular ascorbate peroxidase and catalase, showed divergent patterns. The proteins gradually decreased during olive drupe development, whereas the corresponding transcripts gradually increased (Table 1; Table S2; Table S3). Fruit ripening has been described as a controlled oxidative process whereby H_2_O_2_ and ROS (reactive oxygen species) accumulation are balanced by the activity of cellular antioxidant systems [53], among which major players are catalase and ascorbate peroxidase (APX). The increase in the protein level of ascorbate peroxidase during ripening is well described in tomato [16], [36]. In this context, the identification of catalase and APX as protein spots decreasing during olive drupe development, from 45 DAF to 150 DAF, is surprising but it corresponds to what observed in grape, where both catalase and APX have been found more highly expressed in green tissues than in ripe [54], [55]. As previously shown by Jiménez and co-workers [53], the regulation of the ROS detoxifying enzymes is very complex, iso-enzyme specific and occurring at different levels (transcriptional, post-transcriptional, compartmentalized at subcellular level, etc.). As it appears from the data here presented, a transcription response to oxidative stress is systematically induced during olive fruit development but the protein levels of ROS detoxifying enzymes are subjected to post-transcriptional control. Present understanding of these mechanisms is far from being comprehensive and further data will be needed to better understand this phenomenon.

Among protein spots showing the highest regulation during fruit development, we detected Ole-e-12.01 allergen (Table 1), strongly decreasing in abundance at later stages (Table S2). Allergy to olive is quite common and it is mainly due to the pollen produced in flowers, while allergy to olive fruit and olive oil is less common, though some cases have been also described [8]. The decrease in abundance of allergenic proteins during ripening might offer a possible explanation as to why certain subjects affected by olive pollen allergy can tolerate olive oil. Surprisingly, no transcripts corresponding to this allergen was identified in the transcriptomic investigations, offering the hypothesis that the amount of this important protein is not under transcriptional control.

### Concluding Remarks

To our knowledge, this work is the first large proteomic investigation on olive drupe development. 247 protein spots showing changes in abundance during development were revealed by comparative proteomics. 121 protein spots corresponding to 68 unique proteins were identified and discussed in relation to biochemical processes controlling major fruit development and ripening traits. A number of differentially accumulated protein spots associated to fatty acids biosynthesis and aroma compounds were in fact detected. Comparative proteomics has also provided new insights into fruit photosynthesis, strengthening its pivotal role in oleogenesis. In our view these results shed some light on the developmental process of a non-climacteric fruit to be further investigated in future studies.

## Materials and Methods

### Plant Material

The olive genotype investigated in this study was the *cv*. Coratina, a widely cultivated variety, characterized by a very high phenolic content. Olive fruits were harvested at 45, 110 and 150 days after full bloom (DAF) (Figure 1) from plants of an olive cultivar collection at the experimental farm of the CRA–OLI (Collececco, Spoleto, Perugia) in central Italy (42° 48′ 48″N, 12° 39′ 15″E, 356 m above sea level). Immediately after harvesting, the olive fruits were frozen in liquid nitrogen and stored at −80°C until further analysis. The phenological stages of the fruits at sampling dates correspond to important physiological phases of fruit development, seed development, mesocarp development and the onset of ripening, respectively. Only fruit mesocarp and epicarp have been used for protein extraction.

### Protein Extraction and Quantification

The total protein content was extracted from pooled drupes (at least four drupes for each stage), after pit removal. Protein extracts were obtained by a multi-step protein extraction procedure. In details, 5 g of sample were grinded with liquid nitrogen, using mortar and pestle. The powder was suspended in 50 ml of 20% TCA/water for protein precipitation and removal of phenolics. Precipitated proteins were centrifuged for 30 min at 5000g at 4°C. Precipitated proteins were rinsed twice with 20%TCA in 80% acetone for oil removal. To prevent protein oxidation, pellet was dried under a gentle stream of nitrogen gas. Then, proteins were extracted by using phenol, as described before [9]. Before 2-DE analysis, proteins were desalted and purified with 2D- Clean up kit (GE Healthcare). The concentration of the protein mixtures was estimated by the Popov Amido Black-based method [56] with bovine serum albumin as a standard.

### Experimental Design

For the proteome analyses, an experimental design based on complete sample pooling strategy has been here used [57]. Pooling reduces variability by minimising individual variation and represents an alternative approach to biological replicates in experiments where the interest is not on the individual but rather on characteristics of the population (*e.g*. common changes in expression patterns) [58], [59]. All samples from one developmental stage were pooled together and any replicates were technical replicates of this pooled sample. This approach may be necessary when insufficient material is obtained from an individual [58], [59]. In our case, this approach resulted necessary to overpass limited amounts of proteins obtained from single drupes at 45 DAF (data not shown). For total protein extraction, at least four drupes per stage were here used. To ensure statistical significance for quantitative analyses, four technical replicates were performed for each of the three developmental stages, giving a total of 12 gels.

### Two-dimensional Gel Electrophoresis

200 µg of proteins were dissolved in the DeStreak Rehydration solution (GE Healthcare) and loaded by passive overnight rehydration on Immobile gradient pH 4–7 drystrip, 18 cm. Proteins were focused by using ETTAN IPGphor II system (GE Healthcare), at 20°C with maximum 50 µA/strip and applying 300 V for 5 hr (step and hold), 1000 V (gradient) for 7 hr, 8000 V (gradient) for 3 hr and 8000 V (step and hold) for 1 hr and 10 min, to achieve a total of ∼ 29 KV/hr. After focusing, strips were equilibrated in two steps with equilibration solution (50 mMTrisHCl, pH 8.8, 6 M urea, 2% SDS, 30% glycerol, bromophenol blue) plus DTT (2%) in the first step and plus iodoacetamide (2.5%) in the second. SDS-PAGE was carried out using ETTAN DALT twelve (GE Healthcare) and 12% polyacrylamide gels in 25 mM Tris (pH 8.3), 1.92 M glycine, 1% w/v SDS, with 5W/gel for 45 min and 15W/gel for 4 hr. The protein spots were visualized with SYPRO Ruby fluorescence stain and gel images were taken using a Typhoon laser scanner (GE Healthcare). After the image acquisition with Typhoon, all gels were stained with Colloidal CBB (Coomassie Brilliant Blue) [60] and used for spot picking.

### Analysis of 2-D Gels

Images of the SYPRO Ruby stained gels were imported into Progenesis SameSpots software (Nonlinear Dynamics). Gel images were aligned by automated calculation of ten manually assigned alignment landmark vectors. A fold change of 2, a threshold of ANOVA p-value ≤0.02 and a false discovery rate (q-value) ≤0.01 were chosen as criteria for the identification of differentially accumulated protein candidates. A power >8 was used to define the protein spots chosen for further analysis. Power is a parameter to be used for calculating the minimum sample size required to accept the outcome of a statistical test with a particular level of confidence [61]. Principal component analysis (PCA) was performed using the software GeneSpring (Agilent Technologies).

### In gel-digestion and Protein Identification by MS

Spots of interest were manually excised from Colloidal Coomassie stained gels and subjected to in gel-digestion with trypsin (Promega, porcine sequencing grade). Briefly, gel pieces were washed with 40% ethanol, shrunk by 100% acetonitrile and soaked in 5–10 µl of 12.5 ng/µl trypsin in 50 mM NH_4_HCO_3_ on ice for 45 min, followed by addition of 10 ml 25 mM NH_4_HCO_3_ for rehydration and overnight incubation at 37°C. Peptides were applied to an Anchorchip™ Target (Bruker-Daltonics) as described [62]. A tryptic digest of β-lactoglobulin (5pmol/µl) was used for external calibration. An Ultraflex II MALDI TOF-TOF mass spectrometer (Bruker-Daltonics) was used for peptide mass mapping and peptide fragment ion mapping. MS and MS/MS spectra were acquired in auto-mode using Flex Control v3.0 (Bruker-Daltonics) and processed by Flex Analysis v3.0 (Bruker-Daltonics). MS spectra were acquired in the m/z scan range: 400–5000. Peptide mass maps were acquired in reflectron mode with 500 laser shots per spectrum. MS/MS data were acquired until 1,000–1,600 laser shots were accumulated for each spectrum. An in-house Mascot server (http://www.matrixscience.com), integrated together with BioTools v3.1 (Bruker-Daltonics, Bremen, Germany), was used for database searches in the olive fruit EST database [4] (http://454reads.oleadb.it - 44.299 sequences), in an in-house *Olea europaea* flower EST database (unpublished data –57.600 sequences) and in *Viridiplantae* subset of the non-redundant protein sequence database (downloaded in January 2012), available at the National Center for Biotechnology Information (ftp://ftp.ncbi.nih.gov/blast/db/FASTA/). The following parameters were used for database searches: carbamidomethylation of cysteine and variable oxidation of methionine; one missed cleavage; mass tolerance MS, 80 ppm; MS/MS tolerance 0.5 Da. Filtering of peaks was carried out for known keratin and autocatalytic trypsin peaks; the signal-to-noise threshold ratio was set to 1∶6. For identification by peptide mass mapping, a significant Mascot score (P≤0.05) and at least five matched peptides were required. Identifications based on three matched peptides were accepted if significant scores were obtained for at least one peptide by fragment ion mapping.

## Supporting Information

Table S1 Protein sequences identified with olive in house database.
(XLS)

Table S2 Protein spot accumulation profile during drupe development.
(XLS)

Table S3 Schematic representation of the comparison between protein and transcript profiles. Proteins of interest were grouped according to their function. For each protein, the spot number assigned by the image analysis software(ProgenesisSameSpot - Nonlinear Dynamics) and the schematic evolution during development were reported. For transcripts, the total number of TCs, their list and their schematic prevalent trend during developmentwere also reported. When a prevalent trend was not evident for a given gene function, additional trend graphs were reported.
(XLS)
